# Effects of parental origins and length of residency on adiposity measures and nutrition in urban middle school students: a cross-sectional study

**DOI:** 10.1186/1687-9856-2013-16

**Published:** 2013-10-17

**Authors:** Ranita Kuryan, David Frankel, Barbie Cervoni, Audrey Koltun, Barbara Lowell, Lisa Altshuler, Michael Rosenbaum, Steven P Shelov, Dennis E Carey, Siham Accacha, Ilene Fennoy, Robert Rapaport, Warren Rosenfeld, Svetlana Ten, Phyllis W Speiser

**Affiliations:** 1 Department of Pediatrics, Hofstra North Shore LIJ School of Medicine, Steven and Alexandra Cohen Children’s Medical Center of New York, New Hyde Park, NY, USA; 2 Department of Nutrition, Cornell University, Ithaca, NY, USA; 3 Department of Pediatrics, Infant and Children’s Hospital of Brooklyn at Maimonides, Brooklyn, NY, USA; 4 Department of Pediatrics, Columbia University College of Physicians & Surgeons, New York Presbyterian Hospital, NY, NY, USA; 5 Department of Pediatrics, Winthrop University Hospital, Mineola, NY, USA; 6 Department of Pediatrics, Mount Sinai School of Medicine, NY, NY, USA; 7 Department of Pediatrics, NYU School of Medicine, NY, NY, USA; 8 Current affiliation: Winthrop University Hospital, 259 1st St, Mineola, NY 11501, USA; 9 Current affiliation: New York University School of Medicine, 550 1st Ave, New York, NY 10016, USA

## Abstract

**Background:**

The prevalence of obesity in U.S. has been rising at an alarming rate, particularly among Hispanic, African, and Asian minority groups. This trend is due in part to excessive calorie consumption and sedentary lifestyle. We sought to investigate whether parental origins influence eating behaviors in healthy urban middle school students.

**Methods:**

A multiethnic/racial population of students (N = 182) enrolled in the ROAD (Reduce Obesity and Diabetes) Study, a school-based trial to assess clinical, behavioral, and biochemical risk factors for adiposity and its co-morbidities completed questionnaires regarding parental origins, length of US residency, and food behaviors and preferences. The primary behavioral questionnaire outcome variables were nutrition knowledge, attitude, intention and behavior, which were then related to anthropometric measures of waist circumference, BMI z-scores, and percent body fat. Two-way analysis of variance was used to evaluate the joint effects of number of parents born in the U.S. and ethnicity on food preference and knowledge score. The Tukey-Kramer method was used to compute pairwise comparisons to determine where differences lie. Analysis of covariance (ANCOVA) was used to analyze the joint effects of number of parents born in the US and student ethnicity, along with the interaction term, on each adiposity measure outcome. Pearson correlation coefficients were used to examine the relationships between maternal and paternal length of residency in the US with measures of adiposity, food preference and food knowledge.

**Results:**

African Americans had significantly higher BMI, waist circumference and body fat percentage compared to other racial and ethnic groups. Neither ethnicity/race nor parental origins had an impact on nutrition behavior. Mothers’ length of US residency positively correlated with students’ nutrition knowledge, but not food attitude, intention or behavior.

**Conclusions:**

Adiposity measures in children differ according to ethnicity and race. In contrast, food behaviors in this middle school sample were not influenced by parental origins. Longer maternal US residency benefited offspring in terms of nutrition knowledge only. We suggest that interventions to prevent obesity begin in early childhood.

## Background

The increasing prevalence of obesity in the U.S. and elsewhere has led to a sharp rise in the rate of diagnosis of type 2 diabetes in adolescents over the last 20 years according to the NHANES [1]. This is likely due to multiple factors such as poor diet and/or more sedentary lifestyle. The increase in obesity has been most prominently observed in minority groups such as Native-, Asian-, African-, and Hispanic-Americans [2]. This might be attributable to greater poverty among these groups and genetic/ethnic predisposition [3]. Additional factors include westernization of diet to calorie-dense/low-fiber foods seen with migration, as well as adoption of sedentary lifestyles [4].

There has been an increasing call for prevention in preference to treatment interventions [5]. Once obesity is established, it is difficult to reverse through interventions [6] and it often persists through adulthood, especially if present in peripubertal period or later [7], strengthening the case for early primary prevention. Schools provide a captive audience for such initiatives [8]. Some school-based research studies have focused on interventions in overweight children, primarily through the use of specialized health facilities and after school tutorials [9,10], while others have targeted the whole school population (reviewed in [11]).

Previous studies of Mexican-American adults suggest that diet quality decreases with duration of residence in the United States. Specifically, consumption of fiber, fruit, and vegetables decreases with duration of residence in the United States, whereas consumption of processed foods, refined carbohydrates, and sugars increases [12]. In light of the above we sought to investigate the influence of parental origins on eating behaviors in a multi-ethnic/racial population of urban middle school students.

### Subjects and methods

The ROAD (Reduce Obesity and Diabetes) Study, a 5-year randomized study, was conducted by a research consortium (Columbia University Medical Center, Maimonides Infants and Children’s Hospital, Mt. Sinai School of Medicine, Cohen Children’s Medical Center of New York, and Winthrop University Hospital) that was coordinated through AMDeC (Academy for Medical Development and Collaboration, New York, NY, USA). The ROAD Study examined the prevalence of pre-diabetic phenotypes and the effects of supervised exercise/nutrition education on clinical (adiposity), biochemical (inflammation, lipids, glucose homeostasis) and behavioral risk factors for type 2 DM in a multi-ethnic/racial population of 6th-8th grade students before and after participating in a 14 week school-based health, nutrition, and exercise intervention [13]. Detailed methods for this study have been described elsewhere [8]. The primary objective of our sub-study was to determine how parents’ country of origin, length of residency in the USA, and ethnicity/race affect measures of adiposity and nutrition at baseline. The questionnaires were administered in the first year of student participation in the study, without the benefit of an intervention aimed at improving students’ nutrition and fitness. Because recruitment was not synchronous across all sites, not all subjects were queried about parental origins and length of US residency. The school where most of subjects were recruited was located in Queens, in a heavily Asian area. The primary outcome variables were nutrition knowledge, attitude, intention and behavior, as well as measures of adiposity, specifically, waist circumference z-scores, BMI z-scores, and percent body fat. BMI and waist circumferences were collected at the initial study visit, Z-scores were calculated for BMI using Epi Info (TM) [14], and waist circumference according to Fernandez et.al [15].

Data on parental origin and length of residency in the United States were collected as part of the intake information for each student at their start date in the study. Student nutrition knowledge and dietary behaviors were assessed using modified Hearts N'Parks subscales. Hearts N’Parks is a national, community-based program supported by the National Heart, Lung, and Blood Institute (NHLBI) of the National Institutes of Health and the National Recreation and Park Association (NRPA) [16]. It assesses the student on varied aspects of nutrition, including knowledge (with a maximum score 12 points), behavior (40 points), intention (8 points), and attitudes (7 points). There were a total of 794 middle school-age subjects studied at baseline in the 5 middle schools. Data regarding country of origin data were limited, as this information was not collected from the start in every school. Therefore, 599 subjects had missing country of origin data for one or both parents. Thirteen additional students were missing ethnicity. Therefore, the final sample size in this sub-study was 182 subjects.

The average age was 12.4 years ± 1.0 and more than half of the students were female (60.4%). A plurality of students were of East Asian origin (31.3%). The remaining 68.7% included African American students (14.8%), Caucasians (13.2%), Hispanic (19.2%), South Asian (18.1%) and a small percentage of students identified as “Other” (3.3%).

There were 123 (67.6%) subjects with neither parent born in the US, 17 (9.3%) subjects with one parent born in the US and 42 (23.1%) subjects with both parents born in the US.

Two-way analysis of variance (ANOVA) with an interaction term was used to analyze the joint effects of number of parents born in the US and student ethnicity on each food preference and knowledge score outcome. If the interaction term was non-significant it was removed from the model. The Tukey-Kramer method was used to compute pairwise comparisons to determine where differences lie.

Additionally, analysis of covariance (ANCOVA) was used to analyze the joint effects of number of parents born in the US and student ethnicity, along with the interaction term, on each adiposity measure outcome. If the interaction term was non-significant it was removed from the model. Although not of direct interest, age and gender were considered to be potential confounders of adiposity measures and were therefore included as covariates. The Tukey-Kramer method was used to compute pairwise comparisons to determine where differences lie.

Pearson correlation coefficients were used to examine the relationships between maternal and paternal length of residency in the US with measures of adiposity, food preference and food knowledge.

All statistical analysis was conducted in SAS version 9.3 (SAS Institute, Cary, NC).

## Results

### Waist Z-score

African American students had significantly higher waist z-scores (Table 1) as compared to Caucasian students (P < 0.007), East Asian students (P < 0.0001), and South Asian students (P < 0.001). Hispanic students had significantly higher waist z-scores as compared to East Asian students (P < 0.0001) and South Asian students (P < 0.01). There was no significant association between number of parents born in the US and waist z-score (P < 0.90). The interaction term between number of parents born in the US and ethnicity was not significant and removed from the final model.

**Table 1:** Middle school subjects’ measures of body fat versus number of parents born in the United States and ethnicity/race

Variable *adjusted mean (SEM)	**Waist circumference Z-score**	**BMI Z-score**	**Body fat percentage**
**# of parents born in US**			
None	0.66 (0.16)	0.74 (0.12)	28.76 (0.84)
One	0.52 (0.33)	0.94 (0.25)	28.79 (1.67)
Both	0.54 (0.26)	0.57 (0.20)	26.87 (1.33)
**Ethnicity/race**			
African - American	1.70 (0.27)	1.31 (0.21)	32.46 (1.39)
Caucasian	0.36 (0.30)	0.89 (0.23)	27.15 (1.55)
East Asian	-0.09 (0.25)	0.35 (0.19)	25.58 (1.26)
Hispanic	1.31 (0.23)	0.99 (0.18)	28.87 (1.23)
South Asian	0.18 (0.28)	0.42 (0.22)	27.68 (1.44)
Other	-0.04 (0.54)	0.54 (0.42)	27.11 (2.79)

*Adjusted for age and gender.

### BMI Z-score

The main effect of ethnicity was a significant association with BMI z-score (Table 1, P < 0.009). Specifically, African American students had significantly higher BMI z-scores as compared to East Asian students (P < 0.007) and South Asian students (P < 0.03). There were no other significant associations.

There was no significant association between number of parents born in the US and BMI z-score (P < 0.5). The interaction term between number of parents born in the US and ethnicity was not significant and removed from the final model.

### Percent body fat

The main effect of ethnicity was significantly associated with percent body fat (Table 1, P < 0.001). Specifically, African American students had significantly higher percent body fat as compared to East Asian students (P <0.03). However, this was not related to the number of parents born in the US (P < 0.5).

### Nutrition knowledge

There were no significant associations between nutrition knowledge and number of parents born in the US (P < 0.2) or ethnicity/race (P < 0.5) (Table 1).

### Healthy eating attitude

There were no significant associations between healthy eating attitude and number of parents born in the US (P < 0.4) and ethnicity/race (P < 0.5) (Table 1).

### Healthy eating behavior

There were no significant associations between self-report of healthy eating behavior and number of parents born in the US (P < 0.3) and ethnicity/race (P < 0.4) (Table 1).

### Healthy eating intentions

There were no significant associations between healthy eating intentions and number of parents born in the US (P < 0.5) or ethnicity/race (P < 0.7) (Table 1).

### Length of residency

There was a significant positive correlation between the mother’s length of US residency and nutrition knowledge (Table 2, p < 0.01). There was also a significant negative correlation between mother’s length of residency and healthy eating attitude (p < 0.02).

**Table 2:** Relationships between length of parental US residency versus middle school subjects’ anthropometric measures of body fat, nutrition knowledge, attitudes and behaviors and intentions

	**Mother’s length of residency ρ (P <)**	**Father’s length of residency ρ (P <)**
Waist z-score	-0.038 (P < 0.8)	0.050 (P < 0.7)
BMI z-score	0.004 (P < 1.0)	0.104 (P < 0.4)
Percent body fat	-0.087 (P < 0.5)	-0.157 (P < 0.2)
**Nutrition knowledge**	**0.308 (P < 0.01)**	0.164 (P < 0.2)
**Healthy eating attitude**	**-0.274 (P < 0.02)**	-0.202 (P < 0.09)
Healthy eating behavior	-0.015 (P < 0.90)	0.023 (P < 0.9)
Healthy eating intention	-0.075 (P < 0.6)	-0.016 (P < 0.9)

## Discussion

The primary objective of this substudy was to determine if there were significant correlations of parental origins and ethnicity/race with children’s adiposity measures (such as BMI, waist circumference, and body fat) as well as nutrition knowledge and food-related behaviors. With respect to adiposity measures, the African American group in our population had a higher BMI, waist circumference and body fat percentage, which is similar to recently published findings in our larger data set [17]. Neither ethnicity/race nor parental origins had an impact on nutrition behavior. Nutrition knowledge, but not attitude, improved with mothers’ length of residency. The positive association between maternal length of residency and nutrition knowledge is likely due to media exposure, friends and family, as well as health providers [18]. In many families, mothers are the primary food preparers in the household [19]. A study by Variyam et al reported a significant positive relationship between mothers’ nutrition knowledge and children’s diets; however, this influence decreases as children grow older [20]. The disparity between nutrition knowledge and attitudes among middle school students as related to length of mothers’ US residency highlights the effect of rapid acculturation. Our data seem to agree with those from the National Longitudinal Study of Adolescent Health demonstrating rapid acculturation of overweight-related behaviors, including diet and inactivity among immigrant Hispanic adolescents [21].

As noted above, our study shows that African American adolescents, especially females, had significantly higher waist Z-scores, BMI Z-scores, and body fat percentage in comparison with other racial and ethnic populations. These results confirm previous studies that show disparities between ethnic groups with adiposity measures [22,23]. In addition, parental origins and length of residency did not have a significant influence on our adolescents’ nutrition behavior. Again this may be due to acculturation in early childhood. It has been shown that recent immigration to the United States results in rapid loss of the dietary pattern from parental country of origin [24]. It is also known that younger immigrants tend to change their diets to assimilate to their host country more readily than older ones [25]. As a result, there is a higher risk for obesity associated with length of residence in the United States due to adoption of suboptimal dietary behaviors and sedentary lifestyles, as seen in studies with the Hispanic population [26].

The limitations of our study include the lack of information on parental adiposity measures, as well as socioeconomic status. Parental BMI has been shown to affect their offspring’s dietary behavior as well as weight status [27]. Rates of obesity in most areas of the United States follow a socioeconomic gradient, such that the burden of disease falls disproportionately on people with limited resources, racial-ethnic minorities, and the poor [28].

Childhood obesity may increase adult morbidity and mortality independent of adult BMI and other confounding factors such as family history of cardiovascular diseases, cancer and smoking [29]. Therefore, it is imperative to strive for the prevention of childhood obesity, rather than treat it after it is established or chronic. School-based programs, such as the ROAD Study, represent an appropriate setting for obesity intervention because they offer continuous and intensive contact with children. School infrastructure and physical environment, policies, curricula, and staff all have the potential to positively influence knowledge and lifestyle [30]. Such programs have potential for long-lasting impact if delivered prior to the onset of obesity and its complications. The significance of our findings is that there is rapid acculturation to western diet among adolescents, regardless of parental origins. Our finding that the mother’s length of residency in the USA affects the nutritional knowledge and attitudes of adolescents could influence the way in which we approach teaching young students about nutrition.

## Conclusions

Our main findings are that direct and surrogate physical measurements of adiposity in children differ according to ethnicity and race. In contrast, food behaviors in this cross-sectional middle school sample were not influenced by parental origins. Longer maternal US residency benefited offspring in terms of nutrition knowledge only. We suggest that interventions to prevent obesity begin in early childhood.

## Abbreviations

NHANES: National Health and Nutrition Examination Survey; BMI: Body mass index; CRP: C-reactive protein; ROAD: Reduce Obesity and Diabetes; AMDeC: Academy for Medical Development and Collaboration; NHLBI: National Heart, Lung, and Blood Institute; NIH: National Institutes of Health; NRPA: National Institutes of Health and the National Recreation and Park Association; ANOVA: Analysis of variance; NYC: New York City.

## Competing interests

The authors declare that they have no competing interests.

## Authors’ contributions

RK, PWS, and MR helped to draft the manuscript. PWS, DC, SS, MR, SA, IF, RR, WR, ST participated in the design of the study. DF, BC, AK, BL, and LA participated in collection and abstraction of data. All authors read and approved the final manuscript.

## References

[B1] Cook S Weitzman M Auinger P Nguyen M Dietz WH Prevalence of a metabolic syndrome phenotype in adolescents: findings from the third National Health and Nutrition Examination Survey, 1988-1994 Arch Pediatr Adolesc Med 2003 157 821 10.1001/archpedi.157.8.821 12912790

[B2] Dabelea D Pettitt DJ Jones KL Arslanian SA Type 2 diabetes mellitus in minority children and adolescents: an emerging problem Endocrinol Metab ClinNorthAm 1999 28 709 729 10.1016/S0889-8529(05)70098-0 10609116

[B3] Speiser PW Rudolf MC Anhalt H Camacho-Hubner C Chiarelli F Eliakim A Freemark M Gruters A Hershkovitz E Iughetti L Childhood obesity J Clin Endocrinol Metab 2005 90 1871 1887 15598688 10.1210/jc.2004-1389

[B4] Misra A Ganda OP Migration and its impact on adiposity and type 2 diabetes Nutrition 2007 23 696 708 10.1016/j.nut.2007.06.008 17679049

[B5] Clarke J Fletcher B Lancashire E Pallan M Adab P The views of stakeholders on the role of the primary school in preventing childhood obesity: a qualitative systematic review Obes Rev 2013 [Epub ahead of print] 10.1111/obr.12058 23848939

[B6] Oude Luttikhuis H Baur L Jansen H Shrewsbury VA O'Malley C Stolk RP Summerbell CD Interventions for treating obesity in children Cochrane Database Syst Rev 2009 21 1 CD001872 19160202 10.1002/14651858.CD001872.pub2

[B7] Whitaker RC Wright JA Pepe MS Seidel KD Dietz WH Predicting obesity in young adulthood from childhood and parental obesity N Engl J Med 1997 337 869 873 10.1056/NEJM199709253371301 9302300

[B8] Rosenbaum M Accacha SD Altshuler LA Carey DE Fennoy I Lowell BC Rapaport R Speiser PW Shelov SP The reduce obesity and diabetes (ROAD) project: design and methodological considerations Child Obes 2011 7 223 234

[B9] Collins CE Okely AD Morgan PJ Jones RA Burrows TL Cliff DP Colyvas K Warren JM Steele JR Baur LA Parent diet modification, child activity, or both in obese children: an RCT Pediatrics 2011 127 619 627 10.1542/peds.2010-1518 21444600

[B10] Sacher PM Kolotourou M Chadwick PM Cole TJ Lawson MS Lucas A Singhal A Randomized controlled trial of the MEND program: a family based community intervention for childhood obesity Obesity 2010 18 S62 S68 10.1038/oby.2009.433 20107463

[B11] Zenzen W Kridli S Integrative review of school-based childhood obesity prevention programs J Pediatr Health Care 2009 23 242 258 10.1016/j.pedhc.2008.04.008 19559992

[B12] Sofianou A Fung TT Tucker KL Differences in diet pattern adherence by nativity and duration of US residence in the Mexican-American population J Am Diet Assoc 2011 111 1563 1569 10.1016/j.jada.2011.07.005 21963024

[B13] National Institutes of Health Clinical Trials, Reduce, Obesity and Diabetes (ROAD) http://www.clinicaltrials.gov/ct2/show/NCT00954577

[B14] Dean AG Arner TG Sunki GG Friedman R Lantinga M Sangam S Zubieta JC Sullivan KM Brendel KA Gao Z Epi InfoTM, a database and statistics program for public health professionals 2002 Atlanta: Centers for Disease Control and Prevention http://wwwn.cdc.gov/epiinfo/ (accessed 10/16/13)

[B15] Fernandez JR Redden DT Pietrobelli A Allison DB Waist circumference percentiles in nationally representative samples of African-American, European-American, and Mexican-American children and adolescents J Pediatr 2004 145 439 444 10.1016/j.jpeds.2004.06.044 15480363

[B16] National Heart, Lung and Blood Institute Heart n’ Parks[http://www.nhlbi.nih.gov/health/prof/heart/obesity/hrt_n_pk/index.htm]

[B17] Rosenbaum M Fennoy I Accacha S Altshuler L Carey DE Holleran S Rapaport R Shelov SP Speiser PW Ten S Bhangoo A Boucher-Berry C Espinal Y Gupta R Hassoun AA Iazetti L Jean-Jacques F Jean AM Klein ML Levine R Lowell B Michel L Rosenfeld W Racial/ethnic differences in clinical and biochemical type 2 diabetes mellitus risk factors in children Obesity 2013 21 2081 2090 10.1002/oby.20483 23596082 PMC3766484

[B18] Perez - Escamilla R Himmelgreen D Bonello H Gonzalez A Haldeman L Mendez I Segura-Millan S Nutrition knowledge, attitudes, and behaviors among Latinos in the USA: influence of language Ecol Food Nutr 2001 40 321 345 10.1080/03670244.2001.9991657

[B19] Gualdi-Russo E Manzon VS Masotti S Toselli S Albertini A Celenza F Zaccagni L Weight status and perception of body image in children: the effect of maternal immigrant status Nutr J 2012 11 1 9 10.1186/1475-2891-11-1 23067040 PMC3493294

[B20] Variyam JN Blaylock J Lin BH Ralston K Smallwood D Mother's nutrition knowledge and children's dietary intakes Am J Agric Econ 1999 81 373 384 10.2307/1244588

[B21] Gordon-Larsen P Harris KM Ward DS Popkin BM Acculturation and overweight-related behaviors among Hispanic immigrants to the US: the National Longitudinal Study of Adolescent Health Soc Sci Med 2003 57 2023 2034 10.1016/S0277-9536(03)00072-8 14512234

[B22] Schuster MA Elliott MN Kanouse DE Wallander JL Tortolero SR Ratner JA Klein DJ Cuccaro PM Davies SL Banspach SW Racial and ethnic health disparities among fifth-graders in three cities N Engl J Med 2012 367 735 745 10.1056/NEJMsa1114353 22913683

[B23] Wang YC Gortmaker SL Taveras EM Trends and racial/ethnic disparities in severe obesity among US children and adolescents, 1976–2006 Int J Pediatr Obes 2011 6 12 20 10.3109/17477161003587774 20233157

[B24] Batis C Hernandez-Barrera L Barquera S Rivera JA Popkin BM Food acculturation drives dietary differences among Mexicans, Mexican Americans, and non-Hispanic Whites J Nutr 2011 141 1898 1906 10.3945/jn.111.141473 21880951 PMC3174859

[B25] Pan YL Dixon Z Himburg S Huffman F Asian students change their eating patterns after living in the United States J Am Diet Assoc 1999 99 54 57 10.1016/S0002-8223(99)00016-4 9917732

[B26] Kaplan MS Huguet N Newsom JT McFarland BH The association between length of residence and obesity among Hispanic immigrants Am J Prev Med 2004 27 323 326 10.1016/j.amepre.2004.07.005 15488363

[B27] Burke V Beilin LJ Dunbar D Family lifestyle and parental body mass index as predictors of body mass index in Australian children: a longitudinal study Int J Obes Relat Metab Disord 2001 25 147 157 10.1038/sj.ijo.0801538 11410813

[B28] Drewnowski A Specter SE Poverty and obesity: the role of energy density and energy costs Am J Clin Nutr 2004 79 6 16 14684391 10.1093/ajcn/79.1.6

[B29] Must A Jacques PF Dallal GE Bajema CJ Dietz WH Long-term morbidity and mortality of overweight adolescents: a follow-up of the Harvard Growth Study of 1922 to 1935 N Engl J Med 1992 327 1350 1355 10.1056/NEJM199211053271904 1406836

[B30] Lakshman R Elks CE Ong KK Childhood obesity Circulation 2012 126 1770 1779 10.1161/CIRCULATIONAHA.111.047738 23027812 PMC3785130

